# Genetically predicted elevated circulating 3,4-dihydroxybutyrate levels mediate the association between family Christensenellaceae and osteoporosis risk: a Mendelian randomization study

**DOI:** 10.3389/fendo.2024.1388772

**Published:** 2024-07-17

**Authors:** Dalong Hou, Yang Yang

**Affiliations:** ^1^ Department of Orthopaedics and Traumatology, Shandong Provincial Third Hospital, Shandong University, Shandong, China; ^2^ Jinan No. 3 People’s Hospital, Jinan, Shandong, China

**Keywords:** gut microbiota, osteoporosis, blood metabolites, Mendelian randomization, mediation analysis

## Abstract

**Objective:**

To investigate the impact of gut microbiota on osteoporosis and identify the mediating role of blood metabolites in this process.

**Methods:**

This two-sample Mendelian randomization (MR) study utilized summary level data from genome-wide association studies (GWAS). Gut microbiota GWAS data were obtained from the MiBio-Gen consortium meta-analysis (n=13,266), while osteoporosis summary statistics were sourced from the FinnGen consortium R9 release data (7300 cases and 358,014 controls). Metabolite data, including 1400 metabolites or metabolite ratios, were derived from a study involving 8,299 unrelated individuals. The primary MR method employed was the inverse variance weighted (IVW) method. Reverse MR analysis was conducted on bacteria causally associated with osteoporosis in forward MR. The gut microbiota with the smallest p-value was selected as the top influencing factor for subsequent mediation analysis. A two-step MR approach quantified the proportion of the blood metabolite effect on gut microbiota influencing osteoporosis. IVW and Egger methods were used to assess heterogeneity and horizontal pleiotropy.

**Results:**

IVW estimates indicated a suggestive effect of family Christensenellaceae on osteoporosis (odds ratio(OR) = 1.292, 95% confidence interval(CI): 1.110–1.503, P =9.198 × 10−4). Reverse MR analysis revealed no significant causal effect of osteoporosis on family Christensenellaceae (OR = 0.947, 95% CI: 0.836–1.072, P =0.386). The proportion of the effect of family Christensenellaceae on osteoporosis mediated by circulating levels of 3,4-dihydroxybutyrate was 9.727%. No significant heterogeneity or horizontal pleiotropy was detected in the instrumental variables used for MR analysis.

**Conclusion:**

This study establishes a causal link between family Christensenellaceae and osteoporosis, with a minor proportion of the effect mediated by elevated circulating levels of 3,4-dihydroxybutyrate. Further randomized controlled trials (RCTs) are warranted to validate this conclusion.

## 1 Background

Osteoporosis is one of the most common bone diseases characterized by decreased bone mineral density, leading to weakened bone strength and an increased susceptibility to fractures. Studies indicate that approximately half of adult females and one-fifth of males will experience one or more fragility fractures during their lifetime (1). Osteoporosis involves the disruption of bone homeostasis, where the balance between bone formation and resorption is disturbed (2). Many drugs used to treat osteoporosis are either unsuitable for long-term use or associated with significant side effects (3). Exploring the modulation of gut microbiota as a potential avenue for improving or treating osteoporosis has garnered significant interest.

Existing research suggests a crucial role for gut microbiota in the development of osteoporosis. Xiao, XY et al. (4) in a mouse experiment, found that phytosterols regulate gut microbiota to increase bone mass, exerting an antiosteoporotic effect. Wang, JH et al. (5) conducted a diversity analysis of gut microbiota in osteoporosis and osteopenia patients, identifying statistically significant differences compared to a normal population, indicating that gut microbiota may be a critical factor in osteoporosis development. Seely, KD et al. (6) provided a comprehensive summary of the role of gut microbiota in osteoporosis. Further exploration of specific gut microbiota species and their relationship with osteoporosis holds great significance.

Mendelian randomization (MR) is a research method using genetic mutation data as instrumental variables(IVs) to investigate whether there is a causal relationship at the genetic level, leading to an impact on outcomes within a complex system. Due to its ability to mitigate confounding factors through well-designed studies, MR studies are considered essential tools for studying certain clinical issues. Previous scholars have utilized Mendelian randomization to study factors influencing osteoporosis. Y Yu, XH et al. (7) systematically investigated the impact of 486 blood metabolites on the occurrence of osteoporosis using MR. Moayyeri, A et al. (8) conducted a genome-metabolome-wide MR study on 280 fasting blood metabolites and their association with osteoporosis. Zhang, ZL et al. (9) explored the association between lipid metabolism, methylation aberration, and osteoporosis using MR. However, no study has systematically evaluated the impact of gut microbiota on osteoporosis using the MR method.

This study represents the first attempt to employ Mendelian randomization to explore the influence of gut microbiota on osteoporosis. Additionally, it innovatively delves into the role played by blood metabolites in this causal relationship, contributing to the further development of innovative treatment strategies for osteoporosis.

## 2 Materials and methods

### 2.1 Study design

MR studies require adherence to three core assumptions: ① IVs are correlated with the exposure (Associative assumption); ② IVs are independent of known confounders (Independence assumption); ③ IVs only affect the outcome through the exposure and are not directly associated with the outcome (Exclusion-restriction assumption). We designed the study to meet these assumptions.

**Figure 1:**
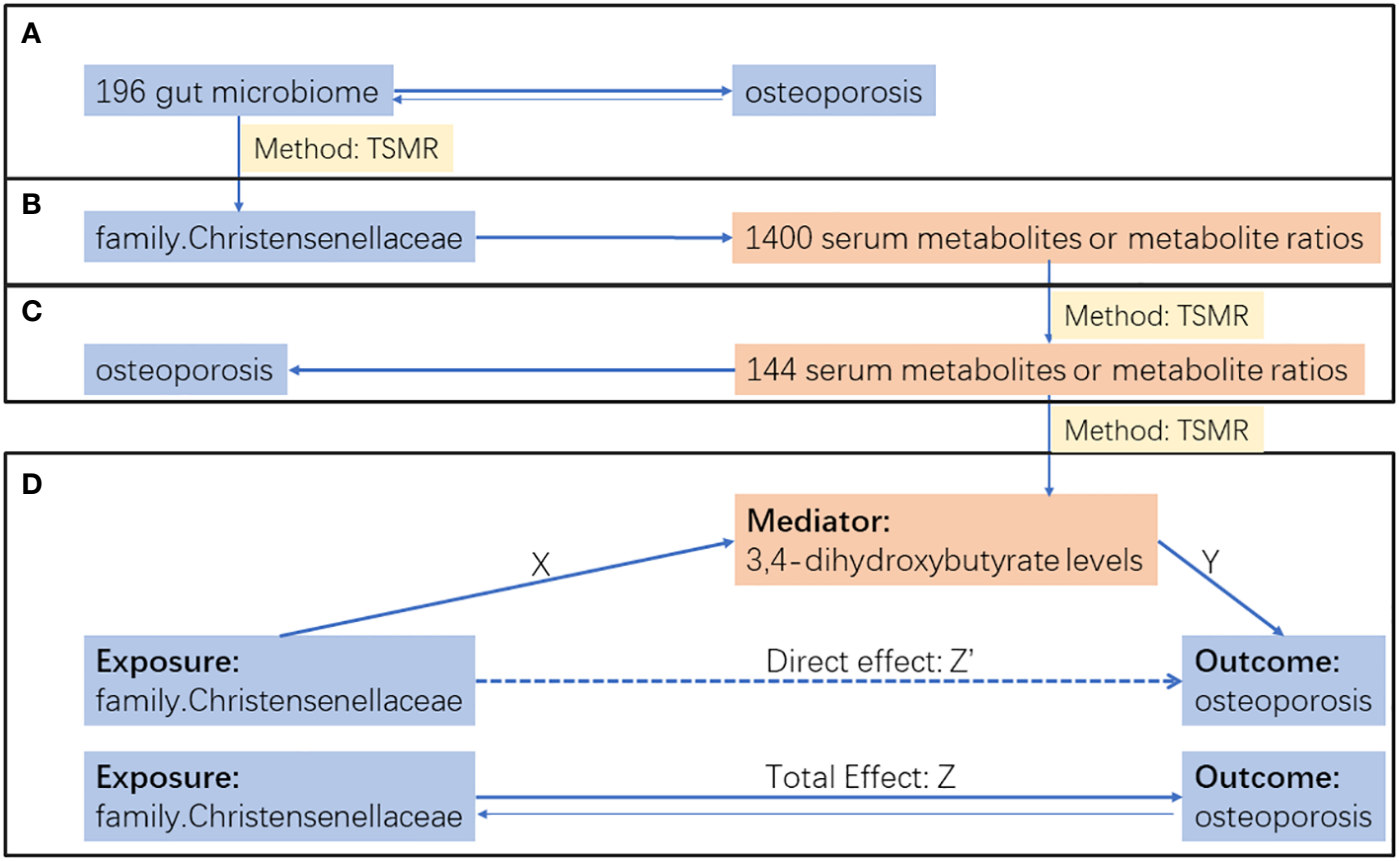
Research flow chart. **(A)** Bidirectional two sample MR analysis between gut microbiota and osteoporosis. **(B)** Two sample MR analysis of top gut microbiota on 1400 serum metabolites or metabolite ratios. **(C)** Two sample MR analysis of serum metabolites or metabolite ratios selected by step B on osteoporosis. **(D)** Mediation analysis. The total effect(Z) was decomposed into: (i) indirect effect using a two-step approach (X × Y) and (ii) direct effect (Z′ = Z – XY). Proportion mediated was the indirect effect divided by the total effect.

We employed a two-step approach to investigate the mediating role of blood metabolites in the process through which gut microbiota influences osteoporosis, as outlined in **Figure 1**. Initially, a bidirectional two-sample MR analysis explored the causal relationship between gut microbiota and osteoporosis. In this analysis, with gut microbiota as the exposure and osteoporosis as the outcome, we excluded results with reverse causation and selected the top microbiota with the smallest P value using the IVW method for subsequent mediation analysis (**Figure 1A**).

In the two-step mediation analysis, the top microbiota was used as the exposure in the first step, and blood metabolites were considered as the outcome (**Figure 1B**). Subsequently, blood metabolites were used as the exposure, and osteoporosis was considered as the outcome in the second step (**Figure 1C**). Results with IVW method p-values less than 0.05 in both steps were selected for the final mediation effect analysis (**Figure 1D**). In all two-sample MR analyses, we excluded results with inconsistent directions in the effects of IVW, MR-Egger, and weighted median methods to ensure the reliability of the conclusions.

### 2.2 Data resource

The summary statistics for human gut microbiome were obtained from the most recent GWAS meta-analysis, including 18,340 participants from 24 cohorts (10). GWAS data for human serum metabolites were sourced from the Metabolomics GWAS Server (http://metabolomics.helmholtz-muenchen.de/gwas/), representing the most comprehensive data on blood metabolites to date (11). GWAS data for osteoporosis were obtained from the FinnGen study, comprising 7300 cases and 358,014 controls (12).

Different consortia provided GWAS data for exposures, mediators, and outcomes, minimizing bias from overlapping samples. As the study used public summary data, no additional ethics approval or consent was required.

### 2.3 Instrumental variables selection

To meet the first requirement of MR analysis, i.e., correlation between instrumental variables and the exposure, SNPs significantly correlated with the exposure (p<1e-5) were selected at the whole-genome level. To avoid linkage disequilibrium and the randomness of interfering SNPs, SNPs with R2 less than 0.001 and genetic distance <10,000 kb were excluded. To satisfy the second MR assumption, i.e., genetic variation is unrelated to potential confounders, the Phenoscanner database was queried to ensure selected SNPs as instrumental variables were unrelated to consistent confounders. A threshold of F value greater than 10 was used for instrumental variable selection to ensure their strength (13).

### 2.4 MR analysis and sensitivity analysis

The primary analysis method employed was the IVW method, which used a meta-analysis approach combined with Wald estimates for each SNP to obtain an overall effect estimate for gut microbiota on osteoporosis (14). The fixed-effect model was used when there was no heterogeneity in the analysis results; otherwise, the random-effect model was employed. IVW method was the main analysis method for evaluating the positivity of MR analysis results. MR-Egger (15) and weighted-median methods (16) were used as complementary methods to IVW. Results inconsistent among the three MR analysis methods led to exclusion. MR-Egger method was also used to assess horizontal pleiotropy, as its intercept can be interpreted as an estimate of the average horizontal pleiotropy of SNPs. The weighted median maintains higher precision compared to MR-Egger in the presence of horizontal pleiotropy. Even if 50% of the genetic variants are invalid instruments, the weighted median provides a consistent estimate.

### 2.5 Mediation analysis

After completing all two-sample MR analyses, a two-step mediation analysis was further conducted to explore the mediating role of blood 3,4-dihydroxybutyrate levels in the process through which family Christensenellaceae influences osteoporosis. The total effect of family Christensenellaceae on osteoporosis comprises direct and indirect effects generated by the mediation factor. In this study, the total effect corresponds to Z in **Figure 1D**, and the indirect effect is represented by XY. The proportion of the mediation effect of 3,4-dihydroxybutyrate was calculated as XY/Z. Confidence intervals were determined using the delta method (17).

## 3 Results

**Figure 2:**
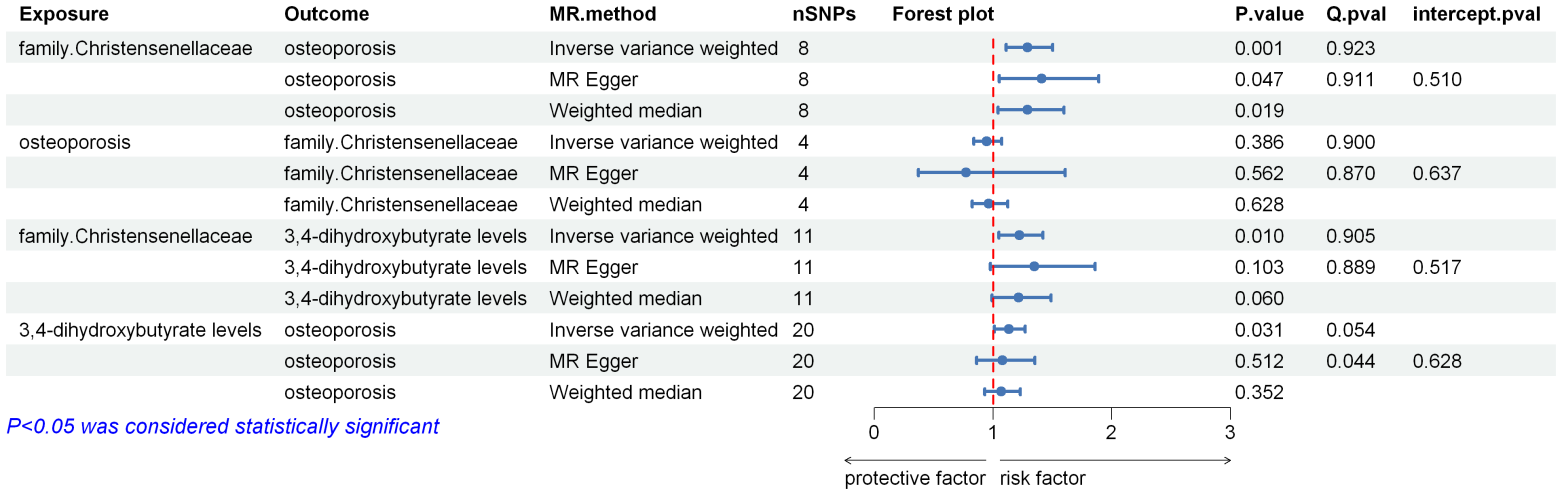
Important outcomes of the two sample MR analysis.

All results of the two-sample Mendelian randomization (MR) analyses and corresponding sensitivity analyses are summarized in **Figure 2**, with the Inverse Variance Weighting (IVW) method as the main statistical approach. In all two-sample MR analyses, the following criteria were applied for result selection: ① IVW method p-value less than 0.05; ② Consistent direction among IVW, MR Egger, and Weighted Median methods; ③ Passing heterogeneity and horizontal pleiotropy tests. It is evident from the results that all MR analyses showed no signs of horizontal pleiotropy or heterogeneity, ensuring the reliability of the findings.

### 3.1 Association of gut microbiota with osteoporosis

The human gut microbiome GWAS data collected included 211 types of human gut microbiota, reduced to 196 after removing unknown microbiota. Using the IVW, MR Egger, and Weighted Median methods, the effects of these 196 gut microbiota on osteoporosis were assessed. After applying predefined filtering criteria, 10 gut microbiota showed statistically significant associations with osteoporosis. The number of SNPs used as instrumental variables (IVs) in these MR analyses ranged from 4 to 18, with F-values all exceeding 10. Among these, the following gut microbiota were identified as risk factors for osteoporosis: phylum Cyanobacteria, family Christensenellaceae, genus *Bifidobacterium*, genus *Eisenbergiella*, genus *Ruminococcaceae*UCG009. Protective effects against osteoporosis were associated with order Actinomycetales, family Actinomycetaceae, genus *Actinomyces*, genus FamilyXIIIAD3011group, genus *Ruminococcaceae*UCG014. Given that family Christensenellaceae had the smallest p-value (IVW OR = 1.292[95%CI,1.110–1.503], P =9.198 × 10−4), and reverse MR analysis did not reveal statistical significance (IVW OR = 0.946[95%CI,0.837–1.072], p = 0.385), it was selected for further analysis.

### 3.2 Association of gut microbiota with serum metabolites or metabolite ratios

Using family Christensenellaceae as the exposure, 1400 serum metabolites or metabolite ratios were subjected to two-sample MR analysis. Each MR analysis involved 11 SNPs, with F-values all exceeding 10. After applying predefined filtering criteria, family Christensenellaceae showed associations with 47 serum metabolites or metabolite ratios. After excluding 8 unknown metabolites, family Christensenellaceae exhibited positive associations with 13 serum metabolites or metabolite ratios and negative associations with 26. These metabolites were included in the subsequent analysis of their associations with osteoporosis.

### 3.3 Association of serum metabolites or metabolite ratios with osteoporosis

Using the serum metabolites or metabolite ratios obtained in the previous step as exposures, and osteoporosis as the outcome, two-sample MR analysis identified 3,4-dihydroxybutyrate levels (IVW OR = 1.133[95%CI,1.011–1.269], p = 0.313) and Alpha-ketoglutarate to succinate ratio (IVW OR = 1.101[95%CI,1.010–1.200], p = 0.029) as risk factors for osteoporosis. The MR analysis results for these two factors showed no signs of horizontal pleiotropy or heterogeneity.

In the two-sample MR analysis with family Christensenellaceae as the exposure and Alpha-ketoglutarate to succinate ratio as the outcome, IVW OR = 0.830[95%CI,0.707–0.974], p = 0.022. This did not align with the direction of the two-step mediation analysis, and therefore, Alpha-ketoglutarate to succinate ratio was not included in further mediation analysis.

In the two-sample MR analysis with family Christensenellaceae as the exposure and 3,4-dihydroxybutyrate levels as the outcome, IVW OR = 1.221[95%CI,1.050–1.420], p = 0.010. This aligned with the direction of the two-step mediation analysis.

### 3.4 Proportion of the association between family Christensenellaceae and osteoporosis mediated by 3,4-dihydroxybutyrate

We conducted a mediation analysis to explore 3,4-dihydroxybutyrate levels as a mediator of the pathway from family Christensenellaceae to osteoporosis. We found an association between family Christensenellaceae and increased serum 3,4-dihydroxybutyrate levels, and serum 3,4-dihydroxybutyrate levels were associated with an increased risk of osteoporosis. Our study indicated that 3,4-dihydroxybutyrate levels accounted for 9.727% of the increased risk of osteoporosis associated with family Christensenellaceae (OR:1.025[95%CI,0.996–1.026]).

## 4 Discussion

In this study, a two-sample MR analysis was employed to investigate the impact of 196 gut microbiota species on osteoporosis. The findings revealed that five gut microbiota species act as protective factors against osteoporosis, while another five act as risk factors, with family Christensenellaceae being notably significant. Further mediation analysis indicated that family Christensenellaceae may increase the risk of osteoporosis through the intermediary effect of serum 3,4-dihydroxybutyrate levels.

Osteoporosis is a common disease influenced by various pathogenic factors, including gut microbiome (18), autophagy (19), abnormal iron metabolism (20), and stress (21). Shasha Song et al. (22) systematically reviewed previous studies on osteoporosis, revealing that the gut microbiome can impact osteoporosis through multiple mechanisms: ①Probiotics could counteract the decrease in osteoblast number and bone mass induced by OVX (glucocorticoids and ovariectomy) by increasing the gene expression of Bmp-2 and Sparc through alterations in the gut microbiome composition.②Probiotics compensate for bone loss by regulating immunity.③Probiotics can prevent OVX-induced bone loss by reducing intestinal permeability and secretion of IL-17, RANKL, and TNF-α from the intestines, inhibiting osteoclastogenesis.

Family Christensenellaceae, belonging to the Firmicutes phylum, is widely distributed in the human gut (23). The genotype of the host is estimated to influence 30%-60% of the variation in the relative abundance of Christensenellaceae (24). Its abundance in the human gut is associated with health (25). Beaumont found a correlation between Christensenellaceae and a lower BMI (26). Considering the existing research showing the correlation between Christensenellaceae and a lower BMI and considering that a lower BMI is a risk factor for osteoporosis, we speculate that Christensenellaceae may impact osteoporosis by influencing human metabolism.

3,4-dihydroxybutyrate is a common metabolic product in the human body, and its levels are elevated in the urine of succinic semialdehyde dehydrogenase deficiency patients (27). Studies by T Minami et al. (28) found that 3,4-dihydroxybutanoic acid gamma-lactone and 2,4,5-trihydroxypentanoic acid gamma-lactone can affect the neural excitability of the lateral hypothalamic area and ventromedial hypothalamic nucleus in rats, brain areas referred to as feeding and satiety centers, respectively. N Shimizu et al. (29) found that injections of 3,4-dihydroxybutanoic acid (2.5 mumol) into the third cerebral ventricle of chronic rats suppressed food intake and single-neuron activity in the lateral hypothalamic area, suggesting that 3,4-dihydroxybutanoic acid may act as an endogenous satiety substance. These studies suggest that 3,4-dihydroxybutyrate may be associated with the feeling of satiety. We can further speculate that an elevated level of blood 3,4-dihydroxybutyrate may lead to increased satiety, thereby reducing the intake of various nutrients, including VitD and calcium, consequently increasing the risk of osteoporosis.

In summary of the above discussion and based on our MR analysis results, we consider that family Christensenellaceae may increase the risk of osteoporosis by elevating serum 3,4-dihydroxybutyrate levels, and previous research findings seem to support this evidence chain.

In our MR analysis, with gut microbiota as the exposure and osteoporosis as the outcome, we found that family Christensenellaceae is a risk factor for osteoporosis. In the MR analysis with family Christensenellaceae as the exposure and blood metabolites as the outcome, we found that the abundance of family Christensenellaceae in the gut microbiota is associated with higher serum 3,4-dihydroxybutyrate levels. Additionally, in the MR analysis with serum 3,4-dihydroxybutyrate levels as the exposure and osteoporosis as the outcome, we found that high levels of serum 3,4-dihydroxybutyrate are associated with an increased risk of osteoporosis. Subsequent mediation analysis revealed that high levels of serum 3,4-dihydroxybutyrate mediate 9.727% of the association between family Christensenellaceae and osteoporosis. Besides the final most significant results, each step of the MR analysis also produced numerous other significant findings, highlighting the close association between gut microbiota, blood metabolites, and osteoporosis. Our findings at each step are, to some extent, supported by previous research, suggesting that our study may provide a valuable summary of existing work.

To the best of our knowledge, this study is the first to use MR methods to investigate the causal relationship between gut microbiota and osteoporosis and the first to explore the mediating role of metabolites in this process. GWAS data for exposure, mediation, and outcome are derived from three different populations, aiming to avoid bias resulting from overlapping study populations. The use of MR methods allows for the effective elimination of confounding factors and reverse causation interference. We conducted sensitivity analyses using MR Egger and IVW methods, effectively addressing issues of horizontal pleiotropy and heterogeneity in the study results. Previous studies have independently explored the relationships between the gut microbiome and osteoporosis, as well as between blood metabolites and osteoporosis. However, comprehensive research investigating the interrelationships among these three factors remains scarce. Thus, our study may serve as a valuable systematic summary and validation of previous findings.

However, this study has certain limitations. The lowest taxonomic level in the exposure dataset was genus, preventing exploration of the causal association between gut microbiota and osteoporosis at the species level. To conduct sensitivity analysis and detect horizontal pleiotropy, more genetic variations need to be included as instrumental variables; therefore, SNPs used in the analysis did not reach the traditional GWAS significance threshold (P < 5×10–8). Consequently, we selected only the gut microbiota with the smallest P-values for further analysis to restrict the overgeneralization of study conclusions. The study population consists of individuals of European descent, limiting the generalizability of the findings to other ethnicities. The study utilized summary-level data, precluding subgroup analysis for more precise conclusions. Further research, using individual-level data, can explore whether the conclusions of this study can be generalized to different populations. Finally, while Mendelian Randomization (MR) analysis can only reflect associations between genetic predispositions, the real-world applicability of our study’s conclusions still requires further exploration through RCTs.

In conclusion, this two-sample MR-based mediation analysis revealed that family Christensenellaceae may increase the risk of osteoporosis by elevating serum 3,4-dihydroxybutyrate levels. Further RCTs are needed to validate this conclusion.

## Data availability statement

The original contributions presented in the study are included in the article/supplementary material. Further inquiries can be directed to the corresponding author.

## Ethics statement

The studies involving humans were approved by Shandong provincial third hospital. The studies were conducted in accordance with the local legislation and institutional requirements. Written informed consent for participation was not required from the participants or the participants’ legal guardians/next of kin in accordance with the national legislation and institutional requirements.

## Author contributions

DH: Conceptualization, Data curation, Formal Analysis, Funding acquisition, Investigation, Methodology, Project administration, Resources, Software, Supervision, Validation, Visualization, Writing – original draft, Writing – review & editing. YY: Conceptualization, Data curation, Formal Analysis, Funding acquisition, Investigation, Methodology, Project administration, Resources, Software, Supervision, Validation, Visualization, Writing – original draft, Writing – review & editing.
